# Analysis of meiotic chromosome structure and behavior in Robertsonian heterozygotes of *Ellobius tancrei* (Rodentia, Cricetidae): a case of monobrachial homology

**DOI:** 10.3897/CompCytogen.v9i4.5674

**Published:** 2015-12-02

**Authors:** Sergey Matveevsky, Irina Bakloushinskaya, Valentina Tambovtseva, Svetlana Romanenko, Oxana Kolomiets

**Affiliations:** 1 N.I. Vavilov Institute of General Genetics, RAS, 3 Gubkin st., Moscow, 119991, Russia; 2 N.K. Koltzov Institute of Developmental Biology, RAS, 26 Vavilov st., Moscow, 119334, Russia; 3 Lomonosov Moscow State University, Leninskiye Gory, 1, Moscow, 119991, Russia; 4 Institute of Molecular and Cellular Biology, Siberian Branch, RAS, 8/2 Av. Acad. Lavrent’ev, Novosibirsk, 630090, Russia; 5 Novosibirsk State University, 2 Pirogov st., Novosibirsk, 630090, Russia

**Keywords:** Meiosis, synaptonemal complex, SC multivalent, hybrid, fertility

## Abstract

Synaptonemal complex (SC) chains were revealed in semisterile intraspecific F1 hybrids of *Ellobius tancrei* Blasius, 1884 (2n = 49, NF=56 and 2n=50, NF=56), heterozygous for Robertsonian (Rb) translocations. Chains were formed by Rb submetacentrics with monobrachial homology. Chromosome synapsis in spermatocytes of these hybrids was disturbed, apparently because of the problematic release of the chromosomes from the SC chains. These hybrids suffer from low fertility, and our data support the opinion that this is because a formation of Rb metacentrics with monobrachial homology within different races of the same species might be an initial event for the divergence of chromosomal forms.

Synaptonemal complex

Robertsonian

## Introduction

Many authors have described the significant impact of chromosomal rearrangements as reproductive barriers in the speciation process (White 1945, Matthey 1951, 1953, Capanna 1982, Baker and Bickham 1986, Sites and Moritz 1987, King 1987, 1993, Vorontsov 1989, Nunes et al. 2011, etc.). W. Robertson first described centric fusions, which join two acrocentrics into one metacentric chromosome, in grasshoppers (Robertson 1916). Named Robertsonian (Rb) translocations, they are widespread among animals, including mammals, and might result in chromosome polymorphism and/or species diversification (Moses et al. 1979, Lyapunova et al. 1980, Searle et al. 1991, Stanyon et al. 2002, Volobouev et al. 2007, and others).

Heterozygosity for the Rb translocations may seriously affect the segregation of chromosomes during meiosis. In heterozygotes, the first division of meiosis will take place without complications only if both acrocentrics, homologous to Rb metacentric arms, move into one pole, whereas the metacentric moves to the opposite pole of the nucleus. Otherwise, if the Rb metacentric and one of the homologous acrocentrics go to the same pole of the nucleus, an inadequate segregation of chromosomal arms will take place and aneuploid gametes will develop (Cappanna et al. 1976, Demin et al. 1983, Redi et al. 1985, Britton-Davidian et al. 1990, Bogdanov and Kolomiets 2007).

A study of the structure and behavior of the synaptonemal complex (SC) is crucial for understanding chromosome synapsis, particularly in heterozygous animals. SC is a skeleton of meiotic bivalents, which form between homologous chromosomes in prophase I of meiosis. The SC analysis allows us to trace all details of the formation of its axial elements, from the beginning of pairing and synapsis at the zygotene, especially synapsis and its correction in heterozygotes for chromosome rearrangements at the pachytene and desynapsis dynamics at diplotene.

The eastern mole vole *Ellobius tancrei* (Cricetidae, Rodentia) is an interesting example of the wide variability of chromosome numbers (from 2n = 30 to 2n = 54 with a stable NF = 56) (Vorontsov and Radzhabli 1967, Lyapunova et al. 1980, 2010, Bakloushinskaya et al. 2013). Unlike *Mus musculus domesticus* Schwarz & Schwarz, 1943 (Capanna et al. 1976, Pialek et al. 2005) and *Sorex araneus* Linnaeus, 1758 (Searle et al. 1991, Wojcik et al. 2003), in which the variability due to Rb translocations is observed over the entire species range, for the eastern mole vole, such an area is limited to a ~150 km territory in the Pamiro-Alay mountains.

Applying comparative chromosome painting (Zoo-FISH) helped to prove the occurrence of non-homologous Rb translocations in different populations of *Ellobius tancrei* (Bakloushinskaya et al. 2010). To evaluate the role of such chromosomal aberrations in the formation of reproductive barriers between intraspecific forms, we investigated the meiotic prophase I of hybrids that cross chromosomal forms with monobrachial homology (MBH) of Rb chromosomes (2n=48 and 2n=50; 2n=50 and 2n=50).

## Materials and methods

### Animals

The specimens studied by us were obtained from laboratory colonies. Hybrid animals were bred in the laboratory from crosses between animals, homozygous for the diagnostic fusions. Animals were kept under standard conditions with free access to food at the facility of the Koltzov Institute of Developmental Biology RAS. Animals were treated according established international protocols, such as the Guidelines for Humane Endpoints for Animals Used in Biomedical Research, and Regulations for Laboratory Practice in Russian Federation, and under the supervision of the Ethics Committee for Animal Research of the Koltzov Institute of Developmental Biology.

As a control we study SCs of two adult males with 2n=54, obtained from two points in Tajikistan: near Miskinobod 38° 39.78’ N; 69° 33.29’ E, 1780 m above sea level, and Panchkotan valley, the left bank of the Sorbo River 38° 45.27’ N; 69° 17.6’ E, 1265 m above sea level. Parental forms for F1 hybrids originated from the right bank of the Surkhob River, close to the airport Garm, 39° 0.28’ N; 70° 17.77’ E, 1310 m above sea level (2n=48), and from the opposite bank of the Surkhob River near the Voydara settlement 38° 58.9’ N; 70° 14.71’ E, 1440 m above sea level (2n = 50). Eight F1 hybrids (2n = 49), five newborn females and three adult males (not younger than 1 year), were investigated. For the second intraspecific crossing we use animals from Voidara (2n=50) and from the Varzob Valley, near the Khodzha-Obi-Garm settlement 38° 53.53’ N; 68° 46.52’ E, 2020 m above sea level (2n = 50). Five adult males of F1 hybrids (2n = 50) were investigated.

### Laboratory analysis

Chromosomes from bone marrow (Ford and Hamerton 1956) were prepared from all animals; tissues of four specimens of all parental forms were used for tissue culturing. Fibroblast cell lines were prepared as previously described (Sitnikova et al. 2007). Full sets of paints derived from flow-sorted chromosomes of the field vole *Microtus agrestis* Linnaeus, 1761 (Sitnikova et al. 2007) were used. FISH was performed according to previously published protocols (Yang et al. 1999). G-banding was carried out for all metaphase chromosomes prior to FISH using trypsin treatments (Seabright 1971).

The suspensions of oocytes and spermatocytes were prepared by the method described by Kolomiets et al. (2010), and spreads were prepared and fixed using the technique of Navarro et al. (1981) with some modifications. Slides coated with poly-L-lysine were used for immunostaining and slides coated with plastic Falcon for electron microscopic study.

The slides were stained with 50% AgNO_3_ in a humid chamber at 56 °C for 3 hours after which they were washed four times in distilled water and air dried. The stained slides were observed by light microscope and suitably spread cells were selected. Plastic circles were cut out with a diamond tap and transferred onto grids. The slides were then examined under a JEM 100B electron microscope.

The slides were washed in PBS. Whole mount SCs were blocked with HB (holding buffer: PBS, 0.3% BSA, 0.005% Triton X-100). The slides were incubated overnight at 4 °C with rabbit polyclonal antibodies against the human lateral element protein SCP3 (Abcam, 15093Ab, UK Cambridge, UK) diluted to a concentration of 1:200 in ADB (Antibody Dilution Buffer: PBS, 3% BSA, 0.05% Triton X-100), human anti-centromere antibodies ACA, 1:200 (Antibody Incorporated, California, USA), and mouse monoclonal antibodies to human mismatch repair protein MLH1, 1:50 (Abcam, Cambridge, UK). The slides were washed in PBS and incubated with goat anti-rabbit Alexa Fluore 488 conjugated antibodies (1:800, Abcam, Cambridge, UK) and goat anti-human Alexa Fluore 546 conjugated antibodies (1:800) at 37 °C for 60 min. The slides were washed with PBS, rinsed briefly with distilled water, dried and mounted in Vectashield with DAPI (Vector Laboratories).

Meiotic cellular suspensions from testes for post-pachytene stages analysis were prepared using the air-drying method (Evans et al. 1964).

The slides were analyzed with an Axioimager D1 microscope CHROMA filter sets (Carl Zeiss, Jena, Germany) equipped with a Axiocam HRm CCD camera (Carl Zeiss), and image-processing AxioVision Release 4.6.3. software (Carl Zeiss, Germany). Images were processed using Adobe Photoshop CS3 Extended. Measurements of autosomal bivalents and their ranking in each cell were made in order to determine relative lengths (MicroMeasure 3.3, Colorado, USA) using the STATISTICA 8.0 software (StatSoft, Tulsa, OK, USA).

## Results

### Heterozygous karyotypes of *Ellobius tancrei*

**Figure 1.**
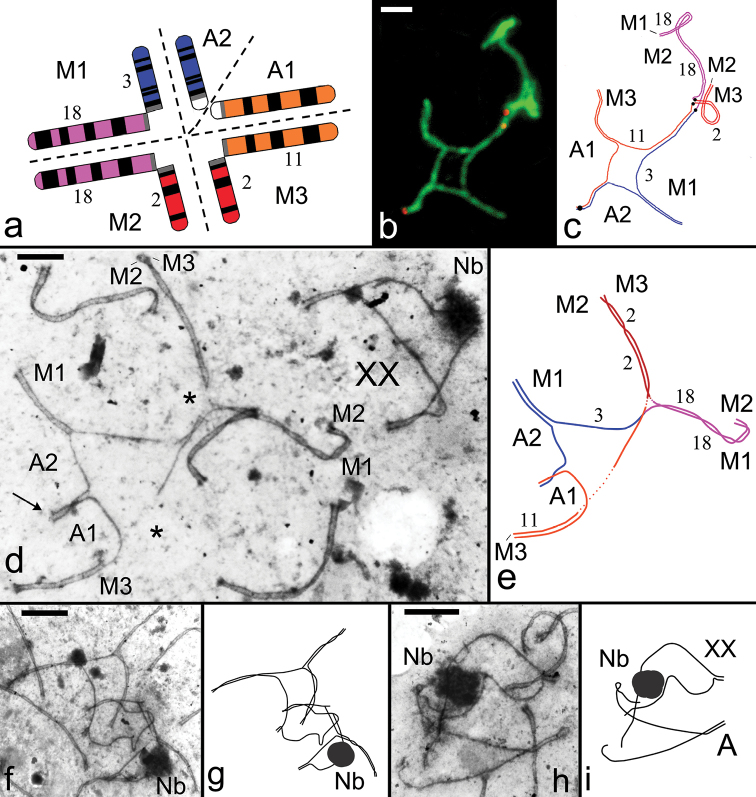
Chromosome synapsis in pachytene spermatocytes of F1 hybrid *Ellobius tancrei* (2n = 49, NF = 56). **a** The scheme reflects a prognosis for chromosome synapsis in prophase I of meiosis [M1=Rb(3.18), M2=Rb(2.18), and M3=Rb(2.11)] **b** Closed SC pentavalent. Immunostaining with antibodies to SC protein 3 (green) and to the centromere ACA (red) **c** The scheme of chromosome synapsis in the structure of SC pentavalent (see Fig. 1b). Black dots mark centromere positions **d** Electron micrograph of spread spermatocyte from F1 hybrid. Closed SC pentavalent is formed from three metacentrics with monobrachial homology (M1, M2, and M3) and two acrocentrics (A1, A2). The arrow shows the fragment of SC between the short arms of homologous acrocentrics. Gaps are marked with asterisks. A sex bivalent (XX) does not associate with the multivalent. Nb – nucleolus-like body
**e** The scheme of chromosome synapsis in the structure of SC pentavalent (see Fig. 1d) **f** A complex case of the association of sex bivalent (XX) **g** The scheme of the association of sex bivalent (XX) with an autosome (see Fig. 1f) **h** An association of sex bivalent (XX) **i** The scheme of the association of sex bivalent (XX) with an autosome (see Fig. 1h). Scale bars: 1 μm (**b, d, i**); 2 μm (**e, f, h**).

Karyotypes of parental forms from opposite banks of the Surkhob River were published earlier (Bakloushinskaya et al. 2010) and were described according to the nomenclature of the *Ellobius tancrei* chromosomes (2n = 54) (Bakloushinskaya et al. 2012). The form with 2n = 48 had six Rb metacentrics [2Rb(2.11), 2Rb(5.9), and 2Rb(3.18)] while the form with 2n = 50, “Voidara,” had four Rb metacentrics [2Rb(2.18 and, 2Rb(5.9)]. There were five Rb metacentrics, three of them with MBH in the F1 hybrids [Rb(2.11), Rb(2.18) and Rb(3.18)] (Fig. 1a).

We identified the Rb translocations in the form with 2n = 50, “Khodza Obi-Garm”, which had four Rb metacentrics [2Rb(4.12) and, 2Rb(9.13)]. F1 hybrids of crossings “Khodza Obi-Garm” with 2n = 50, “Voidara” had four different Rb metacentrics, two of them with MBH [Rb(5.9) and Rb(9.13)] (Fig. 3a).

### Intraspecific hybridization

As a control, we used *Ellobius tancrei* with 2n=54, which is typical for the species. Their fertility was 2.37±0.22 (71 litters, 168 pups). In crossing type I (48 × 50), 24 litters and 62 hybrids F1 were obtained. Litter size was 2.58 ± 1.02. In inbred crosses 11 litters with 16 hybrids F2 were obtained. A litter size was estimated as 1.27±0.47, and the mean litter size was lower comparing the control and parents (p<0.01).

In crossing type II (50 × 50), 35 litters and 106 hybrids F1 were obtained. Litter size was 2.94 ± 0.63. In inbred crosses, 44 litters with 71 hybrids F2 were obtained. A litter size was estimated as 1.61±0.84, with the mean litter size was lower when comparing the control and parents (p<0.01).

### Spermatocytes and oocytes of hybrids with 2n=49

We analyzed a total of 106 spermatocytes of the hybrids (Table 1), including 88 at the pachytene stage. We identified a closed SC pentavalent, formed by three metacentrics and two acrocentrics, at the pachytene stage (Fig. 1a, b, d). A short SC fragment was formed between the pericentromeric regions of the nonhomologous acrocentrics, which are determined by the presence in these chromosome regions of large C-heterochromatic blocks (Fig. 1c, e).

**Table 1.** Synaptic characteristics of multivalents and sex bivalents in the spermatocytes and oocytes of homozygotes and heterozygotes of *Ellobius tancrei*.

Name	Number of cells	Sex (XX) bivalent associations (±SE)	SC multivalent (±SE) (Penta-/tetravalent)		
			With gaps	Closed	Open
*Ellobius tancrei* 2n=54, male	102	0.17±0.03 ^1^	–	–	–
*Ellobius tancrei* hybrid 2n=49, male	106	0.48±0.06 ^1,2^	0.10±0.07 ^3^	0.69±0.09	0.31±0.02
*Ellobius tancrei* hybrid 2n=49, female	59	Sex bivalent behaves as autosomes.	0.29±0.03 ^3,4^	0.62±0.04	0.38±0.03 ^5^
*Ellobius tancrei* hybrid 2n=50, male	94	0,08±0,04 ^2^	0.11±0.04 ^4^	–	0.96±0.06 ^5^

SC – synaptonemal complex, ^1, 2, 3, 4, 5^ Significant difference (p<0.05)

The number of spermatocytes, for which the structure of the sex bivalent and SC multivalent were defined, was less than the total number of cells. This may occur due to weak or partial staining of the axial elements of SC and XX bivalents, which was previously observed in *Ellobius talpinus* (Kolomiets et al. 2010). It may also be caused by strong tensions and numerous gaps in SC structure of multivalent (Fig. 1e). A closed XX bivalent was identified in a significant part of pachytene nuclei (0.84±0.03SE (standard error)). An electrondense nucleolus-like body (Nb) was formed on one of the axial elements of the sex bivalent (Fig. 1). High rates of sex bivalent associations with SC autosomes were observed in the hybrids; the occurrence was significantly higher (p<0.05) than that found in the 54-chromosome form (0.48±0.06 and 0.17±0.03, respectively). This association could have arisen due to heterosynapsis of the axial elements of the sex bivalent with axial elements of the SC bivalents (Fig. 1f, g). As associations, we classified the cases of single and multiple overlapping SC bivalents on the sex bivalent, which occurred in slightly more than half of all associations (Fig. 1h, i).

We studied 59 oocytes in total. According to our observations, in cell suspensions of ovaries, the number of oocytes was lower in hybrids (three to six nuclei per slide) than in ordinary of *Ellobius talpinus* Pallas, 1770 females (40–60 nuclei per slide) (Kolomiets et al. 2010).

**Figure 2.**
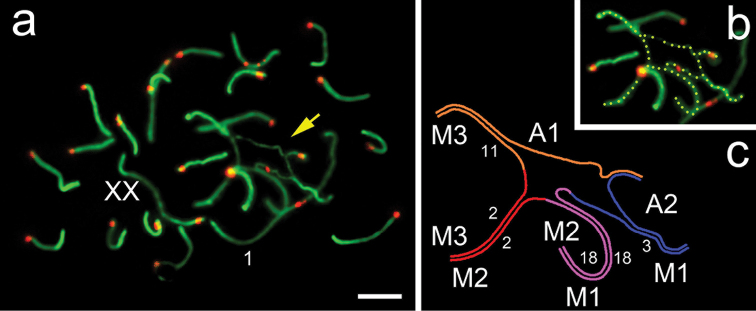
Spreads of oocytes from intraspecific F1 hybrid *Ellobius tancrei* (2n = 49, NF = 56). Immunostaining with antibodies to SCP 3 (green) and to the centromere (ACA, red). The yellow arrow marks the SC pentavalent **a** Early-mid pachytene oocyte: 21 SC bivalents, SC pentavalent, XX bivalent **b** SC pentavalent (yellow dotted line) from Fig. 2a **c** Scheme of SC pentavalent (see Figs 2a, b). The chromosomes forming the SC pentavalent shown in Fig. 1a. Scale bar: 5 μm.

There are 21 autosomal SC bivalents in pachytene oocytes. One SC bivalent was formed by metacentric chromosomes, another SC bivalent appeared due to single pair of non-Rb submetacentrics, and the other 19 SC bivalents were formed by acrocentrics. The rest of chromosomes presented as SC pentavalent and sex (XX) bivalent (Fig. 2a–c).

The XX bivalent that was formed between the two largest acrocentrics was reliably identified via immunostaining of centromeric proteins. Sex chromosomes form the SC, which is indistinguishable from autosomal SCs.

The SC pentavalent was formed by three submetacentrics with MBH, as well as two acrocentrics (Fig. 2a–c), as in spermatocytes (Fig. 1c, e). The SC pentavalent was usually closed due to the non-homologous synapsis of the short arms of the acrocentrics (Table 1). Apparently, nuclear architecture changes in hybrid oocytes, resulting in extension of chromosomes and formation of gaps in the SC axial elements of multivalens (Table 1), similar to what happens in spermatocytes. The formation of SC multivalents in the male and female meiocytes was identical with the only difference in the ability to associate with sex bivalents.

### Spermatocytes of hybrids with 2n=50

**Figure 3.**
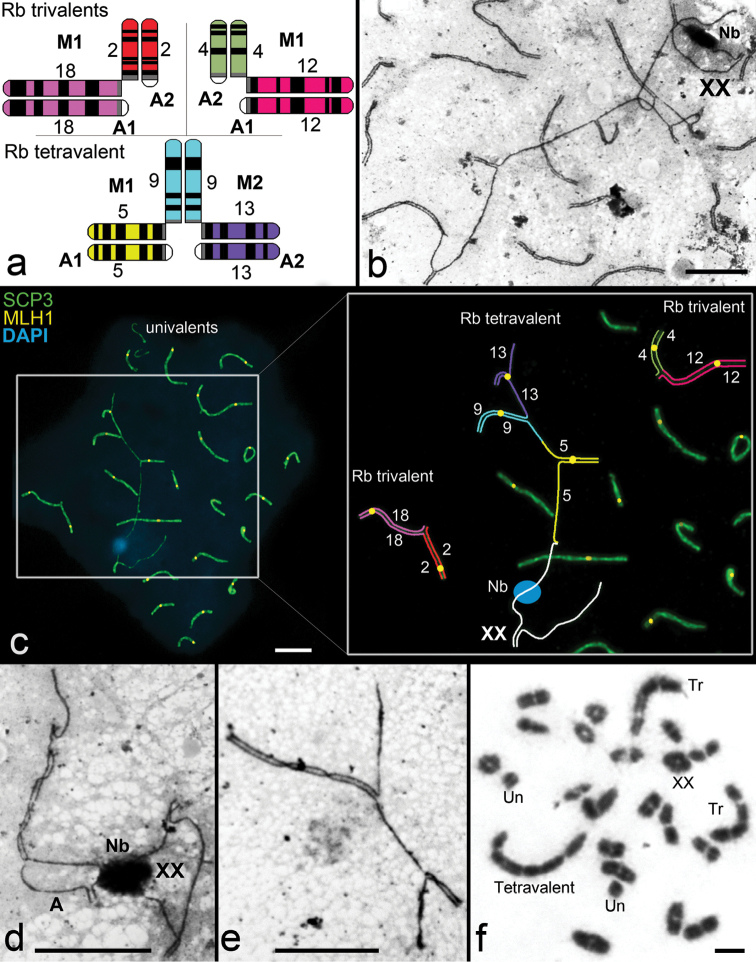
Chromosome synapsis in pachytene spermatocytes and diakinesis/metaphase I cell of F1 hybrid *Ellobius tancrei* (2n = 50, NF = 56). **a** The scheme reflects a prognosis for chromosome synapsis in prophase I of meiosis [Trivalent №1 Rb(2/2.18/18), trivalent №2 Rb(4/4.12/12), and tetravalent Rb(5/5.9/9.13/13)]. M – metacentric, A – acrocentric **b** Electron micrograph of part of the spread spermatocyte. Nb – DAPI-positive nucleolus-like body
**c** Spermatocyte is stained with DAPI (blue). Immunostaining with antibodies to SCP 3 (green) and to MLH1 (yellow) **d** Autosome’s axial element (A) stiking to Nb of sex bivalent (autosome – sex chromosome association) **e** Open SC trivalent **f** Diakinesis/Metaphase I cell showing 18 pairing elements, XX bivalent, two univalents (Un), two trivalents (Tr) and tetravalent. Scale bar: 5 μm.

We analyzed 94 spermatocytes of the F1 hybrid (Table 1). At the pachytene nuclei we distinguished 19 SC and XX bivalents, one SC tetravalent and two SC trivalents. The sex XX bivalent was closed in most of the nuclei (0.87±0.04). Typical SC was formed at the telomeric zones, and usually did not participate in associations with other chromosomes (0.08±0.04). This characteristic was lower compared to the typical *Ellobius tancrei* with 2n=54 (Table 1). One of the few spermatocytes with the autosome – XX association is shown in Fig. 3d. One or two DAPI-positive and electrondense Nbs are formed on one of the axial elements of the sex bivalents (Fig. 3).

The SC tetravalent was usually open (0.96±0.06). It was formed by two Rb metacentrics with MBH and two acrocentrics (Fig. 3). Recombination nodules were detected in synaptic sites of the tetravalent. As a rule, the SC tetravalent did not associate with the sex bivalent, but sometimes we observed end-to-end association of the XX axial elements and the acrocentric bivalents (Fig. 3c).

Each SC trivalent was formed by one Rb metacentric and two acrocentrics (Fig. 3c). Arms of SC trivalents had one recombination nodule. In the most of the nuclei, SC trivalents were closed (0.71±0.03 and 0.63±0.03 for two trivalents, p>0.05). However, the open trivalents (Fig. 3e) sometimes associated with the tetravalents (Fig. 3b).

**Figure 4.**
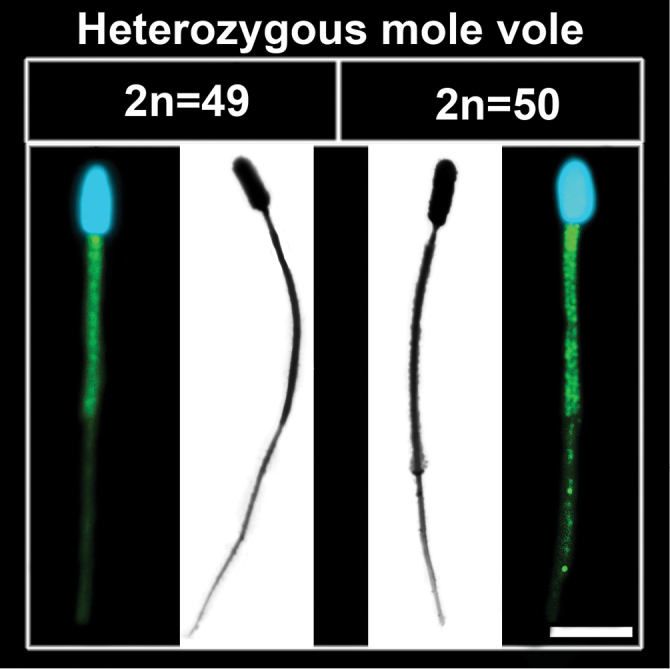
Spermatozoa in meiotic slides of heterozygous *Ellobius tancrei*. Images obtained by AgNO_3_-staining (black and white photo, electron microscopy) and nonspecific immunostaining (color photo, light microscopy). Scale bar: 2 μm.

Small acrocentrics did not undergo synapsis in some pachytene nuclei, and univalents were discovered (Fig. 3c). These data correlated with diakinesis samples: for 92 cells studied, 45 univalents were identified. The diakinesis/metaphase I cell demonstrated 18 pairing elements, XX bivalent, two univalents, two trivalents and tetravalent (Fig. 3f). Mature sperm were frequently seen in meiotic preparations of both types of the heterozygous mole vole (Fig. 4).

## Discussion

Previous studies support the suggestion that the single and multiple Rb fusions may be involved in the process of speciation because accumulation of Rb translocations (including MBH) in different populations of the species can lead to reproductive isolation between them (Gropp and Winking 1981; Baker and Bickham 1986; Hauffe and Searle 1998). The complex compounds, which formed in meiocytes of heterozygous animals, can disturb the synapsis and dissociation of chromosomes during meiosis, leading to the death of spermatocytes, or the formation of unbalanced gametes, and eventually to the hybrid’s sterility.

The impact of simple and complex SC configurations on the progression of spermatogenesis and fertility varied among different groups of animals. Simple and complex SC configurations are well studied in different races of shrews (Narain and Fredga 1998, Pavlova et al. 2008, Matveevsky et al. 2012, etc.). Trivalents in *Cricetulus* hybrids spermatocytes (Matveevsky et al. 2014) and pentavalent in heterozygous *Allocricetulus* spermatocytes led to only partial germ cell death (Gureeva et al. 2015).

In *Mus musculus domesticus*, the impact of Rb translocation in meiotic events and, in general, the formation of gametes, may be insignificant or significant, depending on the number of Rb fusions in the heterozygotes and, therefore, the characteristics and organization of SC chains in the meiotic nucleus. Heterozygous mice with four and seven trivalents had abnormal pairing such as SC trivalent associations with each other and with XY bivalent that might be the cause of infertility (Wallace et al., 2002). Hybrids between monobrachially homologous Rb races of the house mouse from the island of Madeira had two MBH Rb metacentrics, and thereafter in meiosis chromosomes, they formed one chain-of-four configuration (tetravalent). In such semi-sterile hybrids aneuploidy and germ cell death were higher than in homozygous mice (Nunes et al. 2011). It is interesting, that two types of hybrids with MBH Rb metacentrics and SC tetravalents, had a different fertility. Mice heterozygous by Rb(6/6.15/15.4/4) were fertile, but mice with Rb(4/4.6/6.15/15) were sterile (Mahadevaiah et al. 1990). The last type of hybrids demonstrated a high index of asynapsis in SC tetravalents, and a low number of spermatocytes. A study of the SC in *Mus domesticus* hybrids obtained by crossing forms from Campobasso and Cittaducale is of particular interest (Johannisson and Winking 1994). In these hybrids, a closed ring multivalent formed which included 16 Rb metacentrics. In hybrids, obtained by crossing forms from Sicily and Alpie Orobie, an open SC multivalent formed which included 15 Rb metacentrics. Johannisson and Winking (1994) noticed that the formation of closed SC multivalents (SC rings) due to spermatogenic normality. At the same time, the open SC multivalents (SC chains) were often associated with sex bivalent. The mice had a complete spermatogenic breakdown at the spermatocyte I level.

Fertility of lemurs was also variable depending on the number of Rb fusions. Hybrids with three to six SC trivalents were usually fertile. Heterozygous lemurs with eight trivalents in spermatocytes were viable, but had reduced fertility. Hybrids with complex SC configurations, including ones formed by Rb metacentrics that shared monobrachial homology, were sterile (Ratomponirina et al., 1988). In spermatocytes of such hybrids defects of synapsis and the association of sex chromosomes with multivalent were revealed. In spermatocytes of lemurs heterozygous for two MBH, Rb metacentrics found an open SC tetravalent with the terminal association with the XY (Djlelati et al. 1997; Rumpler 2004). The authors suggested that this may lead to a partial loss of meiocytes. The case is similar to associations in *Ellobius tancrei* F1 hybrids with 2n=50. At the same time, Dutrillaux and Rampler (1977) found a small number of spermatocytes with pentavalents in fertile hybrids of lemurs.

Studied heterozygous *Ellobius tancrei* had approximately half the level of fertility in comparison with parental individuals and animals with 2n=54 (see also Lyapunova et al. 1990). Despite the similar parameters of hybrid fertility in spermatocytes, chromosome synapsis with different scenarios was observed. Hybrids with 2n = 49 had an increased level of XX association with other chromosomes, while heterozygotes with 2n = 50 had surprisingly low indices, even in comparison with the animals with 2n = 54. Such associations, a failure of displacement of sex bivalent to the nuclei periphery, and SC configurations are signs of hybrid spermatocytes’ pachytene arrest.

Previously, we have studied other variants of intraspecific hybrids. In the nuclei of spermatocytes of F1 (2n = 44), hybrids from crossing 2n = 54 and 2n = 34 were expected to have six SC bivalents, the sex bivalent, and 10 SC trivalents, each of which was formed as a result of the synapsis of one Rb metacentric and two acrocentrics. However, the expected pattern could be observed only in a single nucleus at the late pachytene. It was found that chromosome synapsis in forming trivalents in most cases was slower than was bivalent synapsis (Kolomiets et al. 1985, 1986, Bogdanov et al. 1986). The process of forming the axial elements slows down in the central part of the Rb metacentrics from SC trivalents, which were highly extended between the points of attachment to the nuclear envelope. There were gaps in the pericentromeric area of the metacentric’s axis until the middle pachytene.

Formation of complex SC configurations (SC chains and rings) in heterozygotes for multiple Rb translocation is accompanied by extended preservation zones of asynapsis in centromeric regions of acrocentrics and metacentrics (Homolka et al. 2007, Mahadevaiah et al. 2008). However, it is known that asynapted chromatin sites undergo transcriptional inactivation, which in fact leads to a block (arrest) of meiosis. According to our observations (unpublished data), the DAPI-positive Nbs of mole vole XX bivalents are chromatin inactivation sites. The apparently unsynapsed site of the SC bivalent anchored to the Nb of XX (Fig. 3d), is a transcriptionally silent region. If the genetically important part of the genome undergoes inactivation, it can lead to germ cell death.

Signs of transcriptional inactivation of asynapted chromatin involving the proteins gamma-H2AX, ATR, and SUMO1 were detected in mice heterozygous for eight Rb translocations. Such inactivation, however, did not lead to disturbances in the formation of the sex body in most of the spermatocytes nuclei (Manterola et al. 2009). This points out the low activity of the pachytene arrest. However, apoptosis of spermatocytes was observed during the metaphase due to defects of the spindle apparatus. The low efficiency of the pachytene checkpoints in relation to the Rb translocations apparently determined the circulation of the Rb metacentrics in natural populations and their roles in the evolution of karyotypes. Perhaps there is low genetic significance of the silenced asynapted centromeric chromatin regions of the SC multivalents, and the SC chains reduce the activity of the checkpoint in the pachytene.

Baker and Bickham (1986) proposed a model of chromosomal speciation by monobrachial centric fusion. Fixation of metacentric chromosomes with homology formed as a result of independent fusion of acrocentric chromosomes can entail reproductive isolation of the population and further speciation due to the accumulation of genetic differences (Capanna 1982, Baker and Bickham 1986). Our data support the possibility of speciation by monobrachial centric fusions in *Ellobius tancrei* as suggested by previous studies for other animals (Capanna et al. 1976, Baverstock et al. 1983, Baker et al. 1985, see review by King 1993). This evolutionary mechanism is likely widely distributed in nature and, along with other chromosomal rearrangements, led to species differentiation.
